# An Air-Filled Bicycle Helmet for Mitigating Traumatic Brain Injury

**DOI:** 10.3390/bioengineering10070762

**Published:** 2023-06-25

**Authors:** Bertrand Mathon, Valentin Duarte Rocha, Jean-Baptiste Py, Arnaud Falcan, Timothée Bergeret

**Affiliations:** 1 Department of Neurosurgery, APHP, La Pitié-Salpêtrière Hospital, Sorbonne University, F-75013 Paris, France; 2 Paris Brain Institute, ICM, INSERM U 1127, CNRS UMR 7225, Sorbonne University, UMRS 1127, F-75013 Paris, France; 3 GRC 23, NeurON-Brain Machine Program, APHP, La Pitié-Salpêtrière Hospital, Sorbonne University, F-75013 Paris, France; 4 GRC 33, Robotics and Surgical Innovation, APHP, Armand Trousseau Hospital, Sorbonne University, F-75012 Paris, France; 5 Bumpair Company, F-94205 Ivry-sur-Seine, Francetim@bumpair.co (T.B.); 6 ICAM Grand Paris Sud, F-77127 Lieusaint, France

**Keywords:** bicycle helmet, concussion, impact testing, impact mitigation, prevention, brain trauma

## Abstract

We created a novel air-filled bicycle helmet. The aims of this study were (i) to assess the head injury mitigation performance of the proposed helmet and (ii) to compare those performance results against the performance results of an expanded polystyrene (EPS) traditional bicycle helmet. Two bicycle helmet types were subjected to impacts in guided vertical drop tests onto a flat anvil: EPS helmets and air-filled helmets (Bumpair). The maximum acceleration value recorded during the test on the Bumpair helmet was 86.76 ± 3.06 g, while the acceleration during the first shock on the traditional helmets reached 207.85 ± 5.55 g (*p* < 0.001). For the traditional helmets, the acceleration increased steadily over the number of shocks. There was a strong correlation between the number of impacts and the response of the traditional helmet (cor = 0.94; *p* < 0.001), while the Bumpair helmets showed a less significant dependence over time (cor = 0.36; *p* = 0.048), meaning previous impacts had a lower consequence. The air-filled helmet significantly reduced the maximal linear acceleration when compared to an EPS traditional helmet, showing improvements in impact energy mitigation, as well as in resistance to repeated impacts. This novel helmet concept could improve head injury mitigation in cyclists.

## 1. Introduction

Traumatic brain injury (TBI) is a significant problem in society that can cause death and long-term disabilities [1,2]. Cycling is a major cause of sports-related TBI both in adults and children, representing nearly 20% of all sports-related brain injuries treated in emergency departments [3,4]. Moreover, with the increasing public awareness of ecology, cycling has become a popular non-motorized means of transportation, exposing cyclists to increased road traffic injuries. A recent report revealed that about 40,000 cyclists die in road traffic injuries each year worldwide [5]. Furthermore, the real number of bicycle crashes is seriously underestimated as most of them seem to be unreported [6].

The head is the most affected body area for severe traumatic injuries among cyclists [7,8]. Bicycle helmets are an effective piece of equipment for head protection during impacts and are effective at reducing TBI risk [9,10]. Contemporary bicycle helmets are made of expanded polystyrene foam with a thermoplastic shell. As shown by previous studies, wearing an expanded polystyrene helmet reduces the risk of skull fracture, penetrating injury, and TBI [11,12,13,14]. Thickness and stiffness, which affect the helmet’s energy absorption efficacy, are the key variables when designing a helmet prototype. Low helmet use levels reported especially in teenagers are mostly related to practical as well as aesthetic considerations [15,16]. These limitations dictate the size, thickness, and design of the helmet and the choice of material constituting the helmet liner. Given its impact mitigation abilities and lightness, expanded polystyrene has become the reference material used by bicycle helmet manufacturers. To further improve safety from different mechanisms of head injury, some research teams proposed to replace the traditional plastic shell with other materials, such as acrylic thermoplastic composite material or coconut shell, while others used collapsible or energy-absorbing structures [9,17].

Recently, bicycle helmets based on airbag technology have been designed [18,19], paving the way for a new generation of helmets. Based on these preliminary works, we developed the Bumpair bicycle helmet, which is filled with pressurized air and uses patented (FR1901909, PCT/FR2020/050360, EP20713723, US17433035) and certified (EN1078/N° 2754/4465/158/11/21/0659, [20]. technology, to increase impact absorption. In this study, we employed an impact test methodology to assess the mitigation of linear acceleration provided by our air-filled helmet concept against a traditional expanded polystyrene helmet.

## 2. Materials and Methods

### 2.1. The Air-Filled Bumpair Helmet

**Figure 1:**
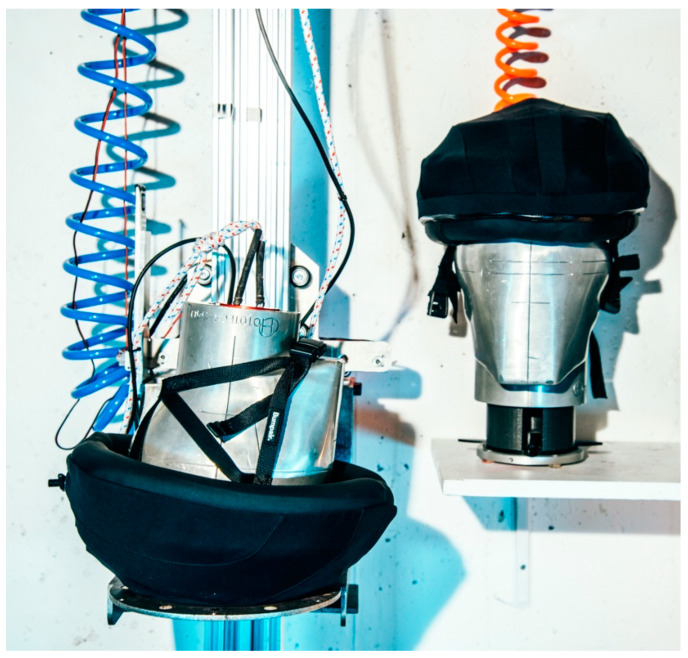
Pressurized Bumpair helmet on a headform.

The Bumpair helmet is a protective helmet comprising an airtight elastic membrane designed to contain gas and a fibrous shell containing said membrane. In the deflated resting state of the helmet, the membrane has dimensions distinct from those of the shell, and the shell containing the membrane can be folded. In the inflated active state of the protective device, the membrane has dimensions corresponding in shape to those of the shell. The shell is designed to retain an expansion of the volume of the membrane beyond the maximum volume of the shell, and said shell is designed to take the form of a helmet. The Bumpair helmet works by encasing pressurized air in a polyurethane pouch inside a polyamide fiber helmet. The polyamide fiber restrains the polyurethane from further deformation and allows it to support high pressure. This surface also creates the shape of the helmet. The pressurized air inside the helmet creates a strong surface tension that allows the helmet to absorb shock and protect the user’s head (Figure 1). The force against the deformation is proportional to the surface in contact with the helmet and the internal pressure. It is important to state that the helmet needs to be inflated to the correct pressure since the protection given directly depends on its inflation levels. To help the user inflate the helmet correctly, a pressure indicator has been added on recent iterations of the helmet to indicate when the helmet is at its correct pressure. Failure to meet the required pressure could be dangerous for the users. The Bumpair helmet is larger than traditional ESP helmets, with an average thickness of 52 mm.

### 2.2. Theory and Study Endpoints

The headform acceleration *g* [m·s^−2^] was used as the primary endpoint to compare the various versions of helmets, particularly the air-filled helmet against a traditional EPS helmet (EPS helmets are not made to withstand more than one impact). The acceleration variable serves as a reference in mechanical impact testing and as a threshold for standard safety tests and is used in the automobile and sports industries and in all domains needing head protection [21]. By definition, it corresponds to the acceleration of gravity at the Earth’s surface and takes the value 9.81 m·s^−2^ in the present study. A G-criterion has been developed and is based on the acceleration measured during an impact test. This criterion can be linked with the head injury criteria (HIC) which is a criterion used in the automobile and sports industries and head protection in general [22]. To meet the European standards (that are detailed in the following paragraph), the G-value must be less than 250. The HIC value is calculated from the linear acceleration endured during the impact and takes into account the duration of this acceleration. For an average adult, an HIC of 1000 results in an 18% probability of a severe head injury, 55% of a serious injury, and 90% of a moderate injury [23].

In the present study, impact testing of bicycle helmets was based on the EN 1078 standard. The EN 1078 standard is a European norm presently used to attain a minimum safety requirement for cyclist helmets before they are authorized to be sold on the European market. The acceleration is measured and must not exceed 2452 m·s^−2^ (250 g with 1 g equal 9.81 m·s^−2)^. The analysis does not take into account the time during which the head is subjected to deceleration. The G-criteria were favored against the HIC value since EN 1078 does not include this criterion and because we did not collect the necessary data to use it.

To compare the protective capabilities of the Bumpair helmet to traditional expanded polystyrene helmets, we choose a competitor’s helmet: the Align II made by Specialized. Both helmets were medium-sized. It is also important to note that the EPS helmets were equipped with MIPS technology. This helmet, like most modern helmets, prevents trauma by using a layer of polystyrene that absorbs the impact and protects the head of the user. It is a highly rated helmet, certified, and widely used. This helmet is a good average budget helmet that represents the other helmets on the market well enough.

### 2.3. Experimental Approach

**Figure 2:**
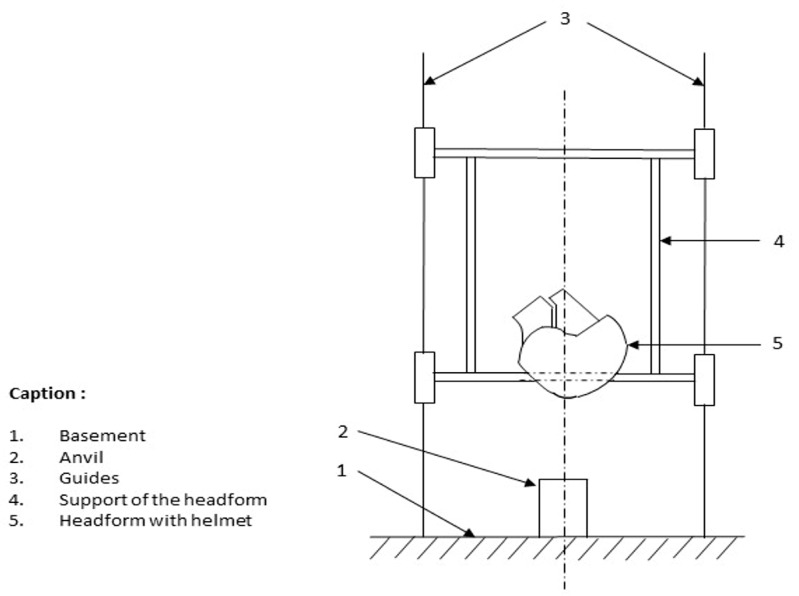
Illustration of a shock test on a helmet define in the document EN1078: helmets for pedal cyclists and for users of skateboards and roller skates (2012), by the European Committee for Standardization [21].

The experimental protocol was based on the EN1078 standard. The impact tests were carried out using certified laboratory-suitable equipment to test the effects on helmets in the event of a fall or collision. All the tests were directed by CRITT, a French laboratory specialized in issuing certificates of compliance with safety requirements. The test benchmark consists of a headform (EN 960 for an M-sized helmet) [24] placed above an anvil. A free-falling guide helps the headform to fall straight. An accelerator was embedded into the headform, and the helmet being tested is positioned on top of the accelerator, as represented in Figure 2. The fall speed was set by the EN 1078 standard: 5.42 m/s for a flat anvil. Theoretically, this speed corresponds to a fall height of 1497 mm. This height was adjusted by measuring the speed at 60 mm before the impact point to take into account the various frictions.

The Bumpair helmets were pressurized at 0.2 MPa, and the pressure was not checked after each impact. All tests were performed at 20 °C. This pressure was determined by Bumpair during the design of the product as the optimal pressure to resist impact events. The first testing phase studied shock repetition on the Bumpair helmet. Multiple helmets were used to increase reliability in the statistical results. We used 4 Bumpair helmets because we did not have a large number of helmets available. The evolution of the state of the helmet was observed over the repetitive tests. The highest acceleration *g* was recorded for every shock. The number of each type of helmet and of repetitive shocks are summarized in Table 1. A second test phase was designed to compare the responses obtained from a single shock on 20 different traditional helmets with the responses of one Bumpair helmet tested 20 times.

### 2.4. Statistics

All statistical analyses were carried out on R with verification and standard test to justify the assertion made in this article. Since we had quantitative variables, we used a parametric Student’s *t*-test to compare them. To study normality, we used the Shapiro–Wilk test, and we used an F test to compare the two variances. We also use Pearson’s product–moment correlation to measure the strength of the association between two variables. With a low number of Bumpair helmets to run the test, we made the hypothesis that a prior impact made on a single Bumpair helmet did not affect the result of a future impact test. It is important to clarify that the helmet does, in fact, deteriorate with the number of uses (as will be shown by the first testing phase), but this deterioration will not be taken into account for the calculus of the second test phase. This hypothesis was very disadvantageous for the Bumpair helmet since the previous test could only deteriorate the absorption capacity of the helmet. This hypothesis was made because we did not have a large number of Bumpair helmets. To prove the efficacy, we calculated the average maximal linear acceleration for each type of helmet.

### 2.5. Ethics

The need for IRB registration was waived because this study does not include human research participants.

## 3. Results

**Figure 3:**
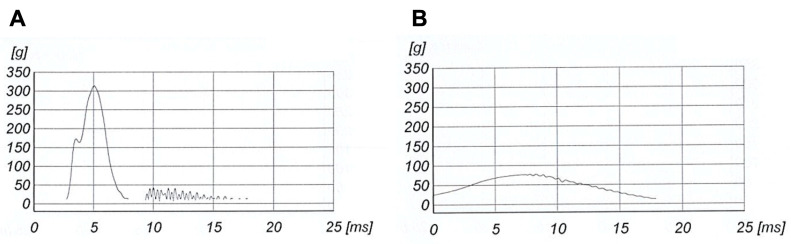
Curves of the acceleration g [m·s^−2^], measured as a multiple of the Earth’s gravity, and plotted as a function of time [ms] obtained during our experiment. Curves recorded for a traditional helmet (**A**) and for a Bumpair helmet (**B**).

**Figure 4:**
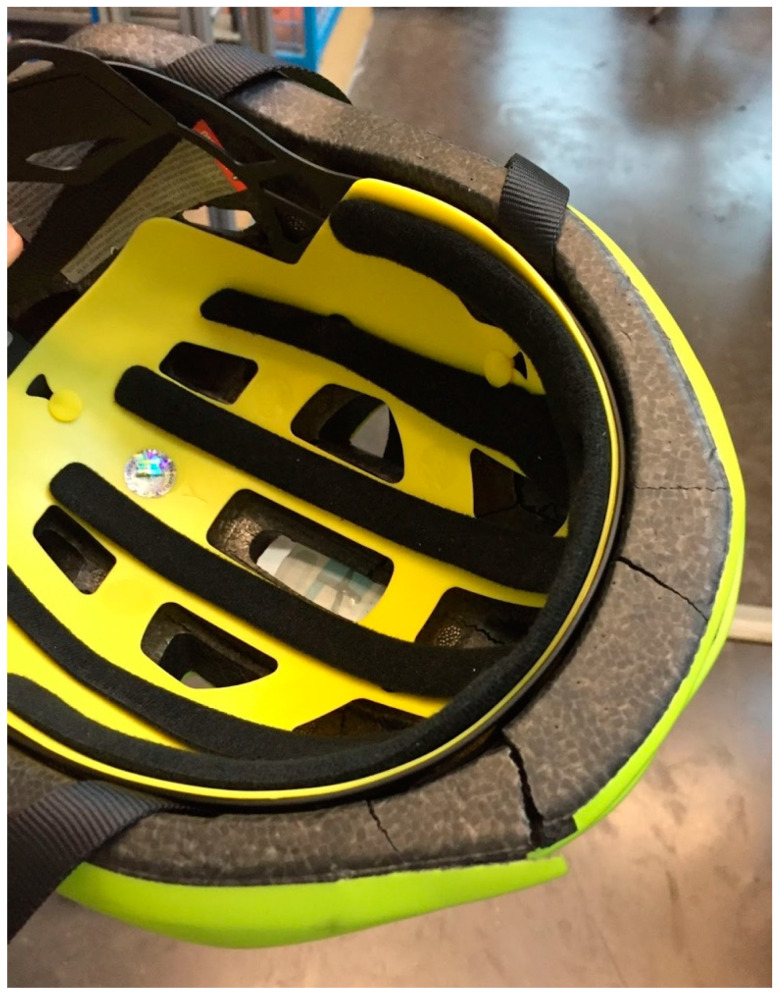
Cracks after several impacts on the traditional helmet. (Traditional EPS helmets are not made to withstand more than one impact).

For each shock, the acceleration recorded by the accelerometer was assessed and the value of the maximum acceleration was extracted. On one hand, the acceleration curves for the traditional helmets all presented a distinctive peak followed by a drastic drop (Figure 3A). It is essential to clarify that the traditional helmet used to obtain this acceleration curve had already been used several times. During the shock event, the traditional helmets deformed and broke, as pictured in Figure 4 (like for Figure 3, it is important to understand that the helmet used in this figure had already been used multiple times). On the other hand, after impact, Bumpair helmets deformed while absorbing the impact energy and regained their original shape after impact. This effect is represented by the curve in Figure 3B, where, after a smooth increase in acceleration, a maximum was reached followed by a smooth decrease in acceleration with almost no oscillations.

**Figure 5:**
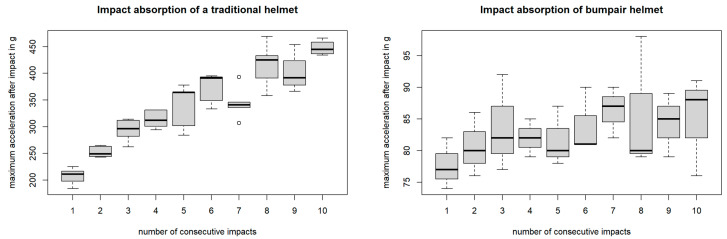
Averaged maximum acceleration [g] values (black lines) +/− standard deviation over 10 repetitive shocks on Bumpair helmet. Dots represent extreme values.

The evolution of the Bumpair helmet responses over the number of impacts is presented in Figure 5. The Bumpair helmets showed a less significant dependence over time, half of the maximum acceleration value of the first shock of the traditional helmets, with less than 100 g. The correlation here was low between the number of impacts and the response of the Bumpair helmet (cor = 0.36 *p* = 0.048). This low correlation supported the hypothesis previously made about the low effect on the Bumpair helmet of the previous impact. The results showed that the Bumpair helmet was more resistant to impacts because its inflatable structure allowed it to better distribute and absorb the impact energy. Further, the traditional helmets reacted less to successive shocks, until they broke. Finally, the Bumpair helmets were inflated by hand, one by one. During this process, the positioning of the air bladders may vary slightly from one helmet to the other. However, despite these variations, the results were consistent and coherent. This showed that human error during the inflation of the Bumpair helmet was compensated for by its design.

**Figure 6:**
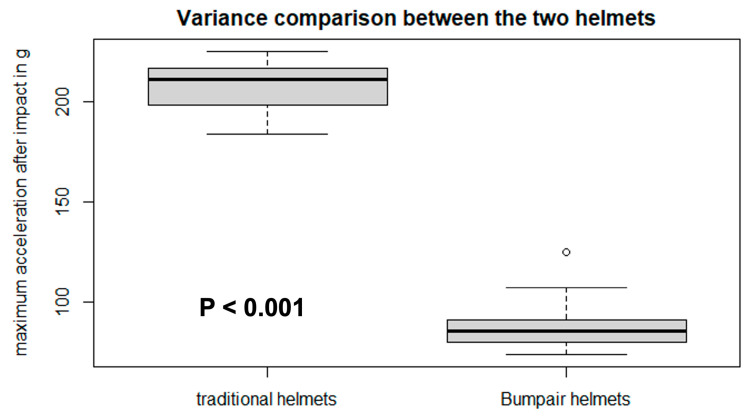
Comparison of the maximum acceleration recorded for the first shock on 20 traditional helmets with the Bumpair helmet. Dot represents extreme value. Black lines represent average values.

The results of the second test phase are summarized in Figure 6. After all the impacts, the Bumpair helmet was still able to absorb more energy than a traditional helmet impacted only once. In fact, the maximum acceleration value recorded during the test on the Bumpair helmet was 86.76 ± 3.06 g (*p* < 0.001) while the acceleration during the first shock of the traditional helmets reached 207.85 ± 5.55 g (*p* < 0.001) representing a minimum factor of 2.25 between them. Further, the acceleration of the Bumpair helmet oscillated between 70 and 110 g while the traditional helmet oscillated between 190 and 220 g during the first impact. Student’s *t*-test allowed us to confirm our hypothesis about the better impact absorption of the Bumpair helmet with a minimum of 112.4 g between the two helmets (114.87; 127.35; *p* < 0.001).

In addition, the traditional helmets exceeded the maximum values allowed by the certification standard after only the second impact since this type of helmet is made to withstand only one impact. This second set of tests further showed that the Bumpair helmet responded quite well to impacts over time since the acceleration did not exceed the maximum allowed value of 250 g after all the tests.

## 4. Discussion

According to the 130 impacts carried out in the test laboratory on the 4 Bumpair samples and 20 specialized samples, this experiment certifies the quality of the Bumpair helmet. The maximum value of its acceleration never exceeded the value required by the standard. The average was always much lower than that of hard helmets.

The Bumpair helmet has already passed the norm EN 1078 and does not fail, even on a curbstone anvil. The injury prevention result on the curbstone type of anvil is even more effective; the maximal linear acceleration recorded is smaller than the acceleration recorded for a flat anvil since the surface of contact is smaller.

The results show an unequivocal difference between the G values obtained on a Bumpair helmet compared to those obtained on a traditional helmet. There is a ratio of 2.25 minimum on the maximum value of G. The properties of the air involved in the protection of the head here are its capacity for deformation, instantaneous absorption, and the distribution of forces throughout the compressed volume. A traditional helmet uses a small area around the impact to protect the user. The kinetic energy is absorbed by the helmet’s material directly above the impact location [25]. The impact greatly damages the helmet to the point that is no longer suitable for protective purposes. A Bumpair helmet uses the entire volume of air encased in its bindings to absorb the shock more effectively. This property allows it to redistribute the impact energy over a longer period resulting in a much lower maximal acceleration for the user’s head. This method to withstand the impact does not damage the helmet and allows it to be subjected to impacts more than once [26].

The Bumpair helmet is different from other products for several reasons, primarily because it uses air to protect the head of the user. The main physical phenomena are simply mechanical. The reaction force during a shock is proportional to the internal pressure of the helmet and the contact surface between the helmet and the object. The main energy lost during a shock is thermal since the compression of the air inside the helmet makes its temperature slightly rise. It is important to note that the absorption capacity varies in the function of the variation in temperature and height. The variation in temperature is taken into account by the norm but not the variation in height.

In addition to offering greater protection, the Bumpair helmet is easy to carry. One of the main aspects of this technology is that it allows the helmet to be compressed and stored easily in the pockets of the user. The helmet is less bulky than traditional helmets and can be taken anywhere without the fear of encumbering the user before or after a ride. Since the best possible helmet cannot protect the user if they decide not to use it [15], it is important to put as many incentives as possible in place to encourage the user to use the helmet on a daily basis [16,27]. To help with these issues, more and more helmets try to offer better aesthetics and designs to promote their use on every ride. The Bumpair helmet also tries to address this matter by featuring a highly customizable protective cover to put on the helmet.

Mathematical theoretical models as well as simulation using 3D modeling and finite element simulation are being developed and will make it possible to anticipate the mechanical response (peak acceleration value, shock duration, HIC value) for new speeds of impacts, new pressures, and also new designs [28,29]. Our results were obtained from laboratory experiments [30]. These results were not validated by clinical tests, as it is not presently possible. In the future, if the Bumpair helmet becomes widely used, it should be possible to test if Bumpair helmet users are less likely to suffer head injuries than traditional helmet users.

The Bumpair helmet is thicker than a traditional ESP helmet. This thickness is needed to permit a lower pressure of usage and to decrease the acceleration withstood by users during impacts. Thanks to the Bumpair technologies, the lower the pressure is, the thicker the helmet needs to be. On the contrary, a thinner helmet would need a higher pressure to withstand the impact. With the actual thickness of the helmet, all the impacts required by the EN 1078 norm can be withstood, with a pressure of 0.18 MPa.

Our results present some weaknesses. First, we only used four Bumpair helmets to perform all the tests. Even if this provides strong clues about the reliability of the results over consecutive impacts, it does not allow us to conclude on the repeatability of these results with all Bumpair helmets. Second, we did not use the HIC in this study. This criterion could have been interesting to study because it takes into account the duration of the impact and since the Bumpair helmet takes longer to absorb the impact than a traditional helmet, the difference in the results between the two could have been even more significant. Third, in this article, the Bumpair helmet was not tested at different temperatures to simulate a range of external variations, which the helmet could be submitted to during its use. The European norm EN 1078 specifies the need to test the helmet at −20 °C and 50 °C. This variation in temperature is particularly important in our case since the Bumpair helmet is a pressurized object, and the internal pressure of the helmet changes significantly with the temperature. For this study, it could have been interesting to see the reaction of the helmet in a wider range of temperatures. Fourth, it is important to specify that traumatic brain injuries are primarily caused by angular acceleration [31]. Since we only studied linear acceleration in this experiment (especially because the experimental test is based on the EN 1078 norm), it could be interesting to study acceleration with a tiling anvil more in-depth. Finally, due to the specificity of the Bumpair technology, the helmet showed a strong bouncing effect. Notably, this effect could have an impact on the user’s cervical spine and has not been studied at present. Since this effect could be more dangerous than previously anticipated, research will be conducted to study in more detail the impact of rebound on the health of users.

## 5. Conclusions

To conclude, we demonstrated that the Bumpair helmet’s capacity to reduce maximal linear acceleration is better than what a traditional helmet provides today. However, these results are only theoretical and do not include head injury criteria, and are not supported by strong clinical studies since the helmet is not widely used for now. In the near future, numerical studies, wider use of the helmet, and other tests could provide a better understanding of the user response to better impact absorption with the Bumpair helmet.

## Figures and Tables

**Table 1:** Types and quantity of helmets used during the testing phase along with the number of shocks made on each one.

Experimental conditions	0.2 MPa; 20 °C; no pressure check between each impact. 5.42 m/s; flat anvil.	
Type of helmet	Bumpair	Traditional
First testing phase		
Amount of helmets	4	N/A
Number of shocks per helmet	10	N/A
Second testing phase		
Amount of helmets	1 of the previous 4	20
Number of shocks per helmet	20	1

## Data Availability

Data are available on request from the corresponding author.
